# New Appliances and Things Medical

**Published:** 1905-09-23

**Authors:** 


### NEW APPLIANCES AND THINGS MEDICAL.

[We shall be glad to receive at our Office, 28 & 29 Southampton Street, Strand, London, W.C., from the manufacturers, specimens of all new preparations and appliances which may be brought out from time to time.]

### NEW ZEALAND DRIED MILK.—DEFIANCE BRAND.

(IMPERIAL DRY MILK CO., WELLINGTON, N.Z., AND LONDON, LANGBOURN CHAMBERS, FENCHURCH STREET, E.C.)

WE are glad to see New Zealand supplying a dried milk powder, the ones we have heretofore examined having been English or Swiss. An obvious advantage of this form of milk is that it can just as well come from the antipodes as from Switzerland, and it was *a priori* to be expected that an agricultural colony like New Zealand would not be long in putting such a product as this on the home market. The powder is accompanied by an analytical report from which it appears that if the powder is treated as directed—namely one ounce of it added to half a pint of water the resulting fluid will be practically identical in chemical composition with a good sample of cow's milk. The powder and the resulting milk is sterile, and we see from an extract of a paper read before the Public Health Congress held in London last summer that it is suggested that milk made according to directions from this powder is more digestible than fresh milk. We are not prepared to deny this, but we should like more evidence of it than we have at present. It seems to us that such a statement can only be substantiated by a series of metabolic experiments on the human subject. The milk, when made according to the directions supplied, forms an exceedingly good substitute, so far as the palate is concerned, for fresh milk, and is certainly equal if not superior to any similar preparation we have examined. We have no doubt that this milk powder is to be distinctly recommended, and is likely to occupy a prominent position in dietetics.

### ICILMA PREPARATIONS.

(ICILMA COMPANY, LIMITED, 142 GRAY'S INN ROAD, LONDON, W.C.)

THREE preparations of this substance have been sent to us, namely Icilma Water, Icilma Soap and Icilma Fluor Cream. Icilma, we understand, is an oxygenised form of Selama water, a natural mineral water coming to the surface by a spring situated at Port aux Poules in Algeria. The only fact concerning the composition of this water and hence of Icilma is that it contains sulphur. Selama water itself, although not very well known in this country, has found in France quite an extensive field of usefulness and is recommended for external application in cases of eczema, and internally in doses of about 2 oz. for indigestion, gout and anæmia. The preparations before us are all for application to skin or mucous membranes, chiefly the former; the icilma water can be applied by means of a vulcanite spray, the fluor icilma cream can be rubbed gently on the skin, and the soap can be used as an ordinary soap. The effect of all these preparations is exceedingly refreshing to the patient and their constant application or use is attended with an improved nutritional condition of the skin.

